# Fatal outcome of a hypersensitivity reaction to paclitaxel: a critical review of premedication regimens

**DOI:** 10.1038/sj.bjc.6601303

**Published:** 2004-01-20

**Authors:** J S Kloover, M A den Bakker, H Gelderblom, J P van Meerbeeck

**Affiliations:** 1 Department of Pulmonology, Erasmus MC, Postbus 2040, 3000 CA Rotterdam, The Netherlands; 2 Department of Pathology, Josephine Nefkens Institute, Erasmus MC, Rotterdam, The Netherlands; 3 Department of Clinical Oncology, LUMC, Leiden, The Netherlands; 4 Department of Respiratory Diseases, University Hospital, Ghent, Belgium

**Keywords:** paclitaxel, hypersensitivity reaction, anaphylaxis, dexamethasone, premedication

## Abstract

Hypersensitivity reactions (HSRs) to paclitaxel are frequently encountered in patients receiving this antitumour drug. Administration of histamine H1- and H2-receptor antagonists and corticosteroids has been shown to reduce significantly the risk of developing an HSR in patients receiving taxanes. In this case report, we describe the fatal outcome of an HSR in a patient receiving paclitaxel despite short-course premedication. The level of evidence supporting the short-course i.v. premedication schedule is challenged, as it is not compatible with the pharmacokinetic properties of dexamethasone.

A hypersensitivity reaction (HSR), defined as any immunological response to a drug resulting in an adverse reaction, is a frequent side effect during paclitaxel infusion (Zanotti and Markman, 2001). Symptoms vary from mild pruritus to anaphylaxis (Weiss *et al*, 1990). The occurrence of HSRs can be influenced by administration of an appropriate premedication. Oral premedication with dexamethasone at a dose of 20 mg given orally at 12 and 6 h before infusion of paclitaxel has been shown to reduce the incidence of paclitaxel-induced HSRs significantly (Weiss *et al*, 1990; Rowinsky and Donehower, 1995; Kintzel, 2001). However, due to logistical factors, short-course premedication with intravenously (i.v.) administered dexamethasone given 30 min prior to paclitaxel infusion has become customary in many centres. Retrospective analyses comparing the effectivity of both prophylactic regimens have been performed. However, outcomes from these studies are inconsistent. In this report, the rationale of short-course i.v. dexamethasone is discussed at the occasion of a fatal anaphylactic event after paclitaxel infusion with the latter premedication regimen.

## CASE REPORT

A 52-year-old male with good performance status was referred for treatment of biopsy-proven non-small-cell lung cancer (NSCLC). The primary lesion was located in the left upper lobe with multiple pleural metastases and thoracoscopic invasion of the mediastinum. The patient was offered palliative chemotherapy consisting of paclitaxel (200 mg/m^−2^) as a 3-hourly infusion followed by carboplatin (area under curve 6) infusion, both as 3-weekly cycles. Premedication consisted of i.v. administered clemastine (2 mg), ranitidine (50 mg) and dexamethasone (10 mg), and was given 30 min prior to paclitaxel infusion. Shortly after the start of the paclitaxel infusion, the patient complained of acute progressive pain in his lower back, breathlessness and chest pain. He developed general distress, followed by cardiac arrest. Cardiopulmonary resuscitation, which included intubation and respiratory support, was started without delay and remained unsuccessful. An autopsy was performed confirming the presence of a stage IV NSCLC extending beyond the parietal pleura into the adjacent soft tissue. Additional findings included mild left ventricular hypertrophy and biventricular dilatation with moderate atherosclerosis of the coronary arteries. Other causes of acute death, such as myocardial infarction, could be excluded. The spleen and liver were congested, consistent with fluid accumulation in the third space.

## DISCUSSION

In this report, we describe the fatal outcome of an acute-onset HSR after paclitaxel infusion, despite administration of a widely accepted regimen of premedication. Postmortem findings after anaphylactic reactions and especially medication-induced anaphylaxis are generally nonspecific and include pulmonary congestion and oedema. Findings indicating an immunological (allergic) cause of death as cutaneous erythema, upper airway oedema and pettecchial haemorrhages are rarely seen (Pumphrey and Roberts, 2000).

Paclitaxel is widely used as antitumour medication in ovarian, breast, non-small-cell lung and other cancers. Owing to the poor insolubility of paclitaxel, the compound requires dissolution in Cremophor EL, a derivative of castor oil.

Although the incidence of HSR after paclitaxel infusion is estimated to be <44 and <10% for mild and severe HSRs, respectively, fatal outcome is rare (Weiss *et al*, 1990). It is known that HSRs predominantly occur during the first 10 min of infusion and are usually restricted to the first two cycles of chemotherapy.

The aetiology of paclitaxel-associated HSR is multifactorial and several mechanisms have been postulated: (A) an IgE-mediated mast-cell degranulation induced by paclitaxel (Weiss *et al*, 1990) or Cremophor EL (Dye and Watkins, 1980; Weiss and Baker, 1987); (B) a non-IgE-mediated idiosyncratic mast-cell degranulation by paclitaxel or by Cremophor EL (Gelderblom *et al*, 2001) and (C) complement activation (Szebeni *et al*, 2001). The rapid and overwhelming onset of the HSR as observed in our patient is not compatible with the natural course of an IgE hypersensitivity reaction and points towards one of the two other mechanisms.

Dexamethasone is a long-acting glucocorticoid with a biologic half-life of approximately 48 h and noticeable onset of biologic activity after several hours (O'Sullivan *et al*, 1997). Dexamethasone strongly inhibits inflammation, especially cellular-mediated immunity and the production or action of the local mediators of inflammation, such as the prostaglandins and lymphokines. Furthermore, dexamethasone reduces vascular permeability and maintains normal vascular responsiveness to circulating vasoconstrictor factors. Standard premedication with dexamethasone at a dose of 20 mg given orally 12 and 6 h prior to paclitaxel infusion has been shown to prevent HSR in most cases (Weiss *et al*, 1990; Rowinsky and Donehower, 1995; Kintzel, 2001). Despite this premedication schedule, grade 3/4 HSR still occur in 1–2% of patients (Kosmidis *et al*, 2002).

However, this treatment regimen requires a good compliance by the patients. To avoid rescheduling chemotherapy schemes due to noncompliance, attention was focused on anecdotal cases in which dexamethasone was administered shortly before paclitaxel infusion. In several retrospective series, 5–20 mg of dexamethasone administered i.v. 3 min before the infusion of paclitaxel showed equal success as the oral premedication of historical cases (Parikh *et al*, 1996; Bookman *et al*, 1997; Micha *et al*, 1998; Markman *et al*, 1999; Kintzel, 2001). These reports are in contrast with a recent report by Kwon *et al* (2002), who retrospectively showed that a single-dose i.v. corticosteroid prophylactic regimen was associated with a significantly higher rate of HSR than the two-dose oral corticosteroid regimen. Bearing in mind the pharmacological properties of dexamethasone, the short-course i.v. premedication schedule given 30 min before paclitaxel infusion is unlikely to result in an adequate level of immunosuppression during the infusion of the drug. This may explain the results of Kwon *et al*. This is confirmed by the observation that the incidence of HSRs is not influenced by the dosage of i.v. administered dexamethasone 30 min before chemotherapy infusion (Koppler *et al*, 2001). It is possible that retrospective analysis and comparison of the study cohort with historical controls bias the observed protective effect of short-course i.v. prophylaxis by dexamethasone. A prospective randomised study in which the oral pre-treatment regimen is compared with the short-course i.v. administration of dexamethasone is preferable.

In conclusion, the fatal outcome of the paclitaxel-associated HSR in the patient illustrates the need for continuous awareness and questions the importance of routine i.v. premedication for paclitaxel administration.
